# Skeletal muscle dissemination in a dog with T‐cell lymphoma

**DOI:** 10.1002/vms3.1060

**Published:** 2022-12-26

**Authors:** Tommaso Magni, Francesca Del Signore, Massimo Vignoli, Rossella Terragni, Alessandro Poli, Francesca Parisi, Michele Sampaolo, Andrea Boari, Arianna Miglio, Paolo Emidio Crisi

**Affiliations:** ^1^ Veterinary Clinic Pet Care Bologna Italy; ^2^ Department of Veterinary Medicine Veterinary University Hospital University of Teramo Teramo Italy; ^3^ Department of Veterinary Science University of Pisa Pisa Italy

**Keywords:** canine lymphoma, computed tomography, muscle metastasis, neoplastic invasion, non‐epitheliotropic cutaneous T‐cell lymphoma, oncologic staging

## Abstract

A 5‐year‐old spayed female American Staffordshire was referred for weakness, reluctance to move and distension of the abdomen. Three weeks before, the dog underwent surgery for excision of a nodular mass suspected to be a non‐epitheliotropic cutaneous T‐cell lymphoma (NE‐CTCL). Computed tomography revealed heterogeneous enhancing mesenteric masses and nodular lesions of soft tissue density, and infiltration of the abdominal muscular wall. Moreover, a pattern of diffuse muscle nodules in the skeletal muscles was visible, with lesions showing homogenous, heterogeneous or ring enhancement. Necrosis was histologically observed and these lesions were infiltrated by CD3‐positive and CD20‐, CD79a‐ and Iba1‐negative neoplastic lymphocytes. On the basis of the immunopathological features metastatic NE‐CTCL was suspected. Skeletal muscle metastasis has been rarely reported in small animals and this case report further confirms that this possibility should be considered in dogs with lymphoma.

## Graphical abstract

Skeletal muscles metastases are considered quite rare in small animals.

When it occurs, muscle metastases typically involve paravertebral muscle and hind limb.

Whole‐body CT is of paramount importance in patients affected by neoplasms with potential systemic metastases.

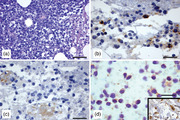

## 1 CASE PRESENTATION

A 5‐year‐old spayed female American Staffordshire Terrier was referred for 14 days of weakness, reluctance to move and distension of the abdomen. The dog had undergone surgery 3 weeks before for the excision of a 2 cm nodule close to the right shoulder. At the time of the excision, the nodule was the only complaint and the onset of the clinical signs was noted by the owner 1 week after the surgery. Histopathology of the cutaneous nodule revealed a proliferation of medium size lymphoid cells, characterised by round nuclei with condensed chromatin and scant cytoplasm on the superficial dermis and the perifollicular areas, without epitheliotropism. Immunohistochemical examination performed on formalin‐fixed and paraffine embedded (FFPE) tissue revealed an intense positive reaction for anti‐CD3 antibody. Based on the proposed criteria (Day, 1995; Kondo et al., 2019), a suspected diagnosis of non‐epitheliotropic cutaneous T‐cell lymphoma (NE‐CTCL) was made.

At the time of the referral, the physical examination revealed a poor body condition score of 3/9, enlarged abdomen and marginal inguinal lymph adenomegaly. No other abnormalities were detected during the physical examination. The dog underwent complete blood count, serum chemistry and urinalysis. The only abnormality identified on haematology, serum biochemistry and urinalysis was mild leukopenia (5.4 × 10^3^/μl, reference interval 6.0–17.0) with no evidence of atypical lymphocytes on blood smear.

### 1.1 Imaging findings

Echocardiography and thoracic radiographs were within normal limits, ultrasonography (Esaote Mylab 70 XV, Esaote Biomedica, Genova, Italy) confirmed the presence of a large amount of abdominal fluid. Following abdominocentesis, this was later identified as a protein‐rich transudate (total protein 3.3 g/dl; specific gravity 1008) with a low cellular content (2.3 × 10^3^/μl) characterised by macrophages, neutrophils and a small number of lymphocytes without signs of atypia. In the caudal abdomen, mesenteric heterogeneous and ill‐defined hypoechoic nodules were visible, but the fine needle aspiration cytology performed was unconclusive.

**FIGURE 1:**
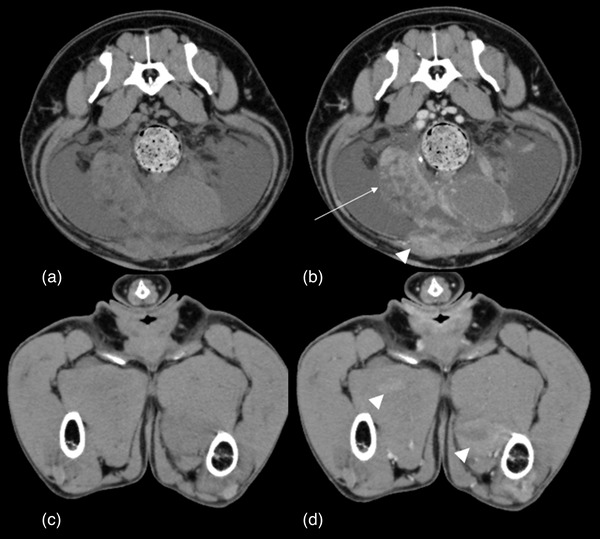
Pre‐ and post‐contrast CT images of the caudal abdomen and proximal hind limbs. In a and b, peritoneal effusion and an heterogeneous nodules (arrow) are visible. Muscular nodules (arrowhead) in the abdominal wall, inguinal, and flexors muscles of the legs are visible, with homogenous or ring enhancing patterns visible only in the post‐contrast study (c and d).

**FIGURE 2:**
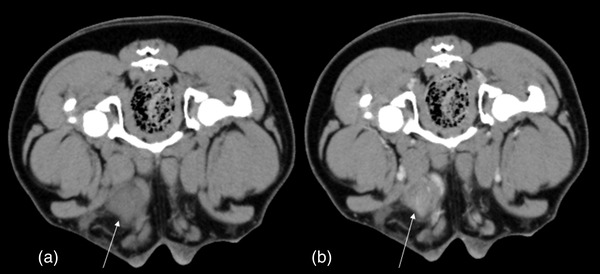
Pre‐ and post‐contrast CT images at the level of the inguinal regions. A nodule with heterogeneous contrast enhancement (arrows) is visible.

Computed tomography (CT) imaging was done under anaesthesia with the dog in sternal recumbency. Pre‐ and post‐contrast CT of the head, thorax and abdomen were performed (GE Optima 540, GE Healthcare, Milwaukee, WI, USA) using 600 mg/kg of intravenous Iopamidol (Iopamiro, Bracco Imaging S.p.A., Milan, Italy) intravenously. CT revealed a marked abdominal effusion, with heterogeneous enhancing mesenteric masses on either side sides of the urinary bladder, of mixed fat and soft tissue density, and multifocal nodular lesions were evident within the abdominal wall musculature. In the right inguinal region, a 3 cm heterogeneous nodule was present located in the area where the external, internal obliques and transverse muscle of the abdomen come together. Throughout the skeletal muscles there were multifocal nodular lesions of various size, ranging from 5 mm for the smallest diameter to 3 cm for the largest diameter, showing homogenous, heterogeneous or ring enhancement (Figures 1a–d and 2a and b). The muscular nodules were present in the paraspinal muscles of the neck, thorax and lumbar spine, and in the right psoas muscle. In addition, there were nodules in the quadriceps muscles in the right leg. Lymph adenomegaly of the right sternal (2 cm cr‐cd, 1.3 cm lateral, 1.3 cm dv, diameters), mediastinal (1.8 cm crcd, 1.1 cm lateral, 1.3 dv, diameters), medial iliac (3.2 cm crcd, 1.5 cm lateral, 1 cm dv, diameters) and inguinal (1.5 cm crcd, 1 cm lateral, 1.3 cm dv, diameters) lymph nodes was present.

Other radiographic findings included osteoarthritis of stifles, disc protrusions and ventral vertebral spondyloses.

An ultrasound‐guided tru‐cut biopsy with a 14G semi‐automatic needle was taken from the inguinal muscle lesions.

### 1.2 Histopathology and immunohistochemistry

The muscle biopsy was formalin fixed and routinely paraffin wax embedded. Four‐micron‐thick sections were stained with Haematoxylin and Eosin, Goldner's Trichrome stains. Tissue sections were further labelled immunohistochemically for B lymphocytic cells using anti‐CD20 antibody (Degl'Innocenti et al., 2019) and anti‐CD79a antibody (Fernandez et al., 2005) and T lymphocytic cells by an anti‐CD3 antibody (Fernandez et al., 2005) and histiocytic cells by anti‐Iba1 antibody (Ohara et al., 2019), following a routine protocol. Briefly, 4‐μm‐thick sections were de‐waxed in xylene, hydrated throughout a graded series of ethanol and rehydrated in deionised water. Antigen retrieval was performed with citrate buffer pH 6.0 in a microwave oven. After exhausting endogenous peroxidases activity with Peroxidase blocking solution® (Dako, Glostrup, Denmark), non‐specific reactions were blocked by incubating each section with two drops of Ultra V‐block® (ThermoFisher Scientific, Waltham, Massachusetts, USA). The primary antibodies used were a rabbit polyclonal anti‐CD3 antibody (clone A0552, dilution 1:200; Dako, Glostrup, Denmark), a rabbit polyclonal anti‐CD20 antibody (clone RB‐9013‐PO, dilution 1:100 Thermo Scientific, Chesire, UK), a mouse monoclonal anti‐CD79a antibody (Clone HM/47A9, dilution 1:100 ThermoFisher Scientific; Waltham, MA, USA), a rabbit polyclonal anti‐Iba1 (ab153697, dilution 1:300 Abcam, Cambridge, MA, USA) and a mouse monoclonal anti‐Ki‐67 antigen antibody (Clone MIB‐1, dilution 1:50; Dako, Glostrup, Denmark). After washes sections were incubated with a universal polyvalent biotinylated antibody (Horse anti‐mouse/rabbit IgG RTU, Vector Lab, Burlingame, CA, USA) for 15 min. After washing, streptavidin‐peroxidase complex (Vector Lab. Burlingame, CA, USA) was incubated for 15 min. Peroxidase activity was revealed by incubation for 10 min in 3,3′‐diamonibenzidine tetrahydrochloride (ImmPACT DAB Peroxidase Substrate Kit®, Vector Labs inc., Burlingame, CA, USA) and blocked with deionised water. Finally, sections were counterstained with Mayer's Haematoxylin, dried and covered with cover slips. Negative controls were performed by replacing the primary antibody with an unrelated mouse isotype matched control monoclonal antibody (clone MA5‐14453; TermoFisher Scientific, Rockford, IL, USA) and a rabbit polyclonal anti‐toxoplasma antibody (Neomarkers, Fremont, CA, USA).

**FIGURE 3:**
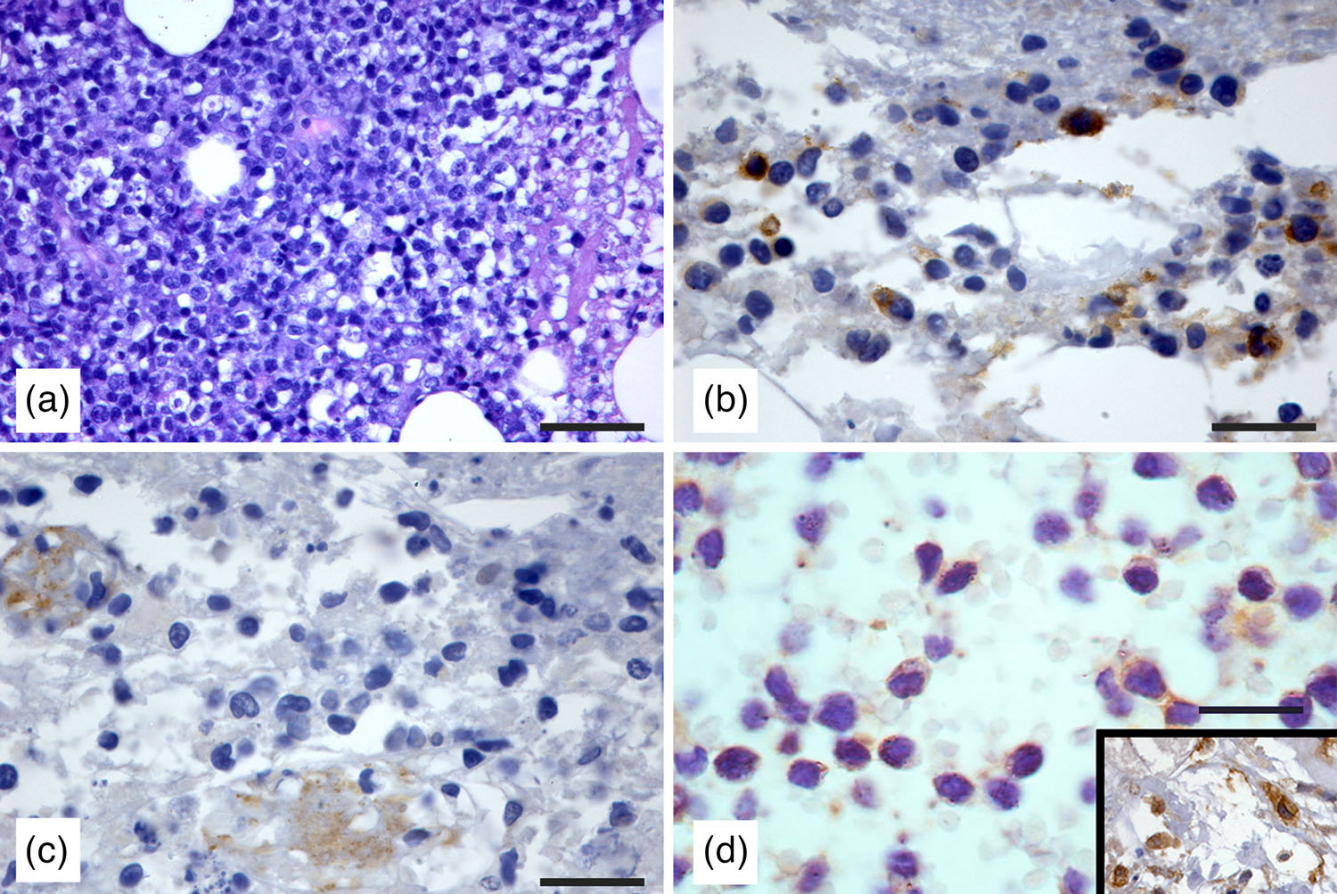
Histological and immunohistochemical features of the muscle biopsy from a dog with a non‐epitheliotropic‐ cutaneous T‐cell lymphoma. (a) Necrotic muscle tissue was infiltrated by neoplastic lymphoid cells scattered within necrotic area were CD3‐positive (Haematoxin‐Eosin, bar = 200 μm). (b) CD3‐positive neoplastic lymphoid cells (IHC anti‐CD3 primary antibody, bar = 50 μm). (c) Neoplastic cells scored negative for CD79a (IHC anti‐CD79a primary antibody, bar = 50 μm). (d) Neoplastic T lymphoid cells were Iba1‐negative, while macrophages scattered throughout the connective tissue scored positive. Detail of positive macrophage staining is shown in the inset (IHC anti‐Iba1 primary antibody, bar = 50 μm).

Histological examination of muscle biopsies demonstrated that necrotic muscle tissue was replaced by medium size lymphoid cells characterised by irregularity of cell size, nuclei with finely reticulated chromatin in the lager cells and denser in the smaller ones, poorly visible nucleoli, nuclear indentation and scanty cytoplasm (Figure 3a). The lack of interlacing fibrous trabeculae was testified by the negative Goldner's Trichrome stain. Mitoses were not prominent (4 mitoses/10 high power fields). Immunohistochemical examination using formalin‐fixed tissue revealed that neoplastic cells scored CD3‐positive (Figure 3a and b) and ‐negative for CD20, CD79a (Figure 3c) and Iba1 (Figure 3d). On the basis of the histopathological and immunopathological features an infiltration secondary to a cutaneous T‐cell lymphoma was suspected. Neoplastic T‐lymphocytes expressing Ki‐67 antigen were 8%.

### 1.3 Therapy and follow‐up

Since the patient's clinical condition was progressively deteriorating, a rapid induction protocol with L‐Asparaginase at 400 IU/kg subcutaneously (SC) was settled for the first week with improvement of the patient's clinical condition, characterised by a marked reduction of the abdominal effusion. For the following 2 weeks, chemotherapy was given using a COP protocol. Vincristine at 0.75 mg/m^2^ intravenously (IV) was given for the first week followed by cyclophosphamide at 300 mg/m^2^ (divided into 4 days) per os (PO). Concurrent prednisolone therapy was given at an initial dose of 20 mg/m^2^ followed by gradual tapering. The patient was asymptomatic with prednisolone 10 mg/m^2^ every other day for 42 days; however, an acute and progressive lameness was reported 1 week later. A rescue chemotherapy protocol with CCNU (lomustine) at 80 mg/m^2^ PO every 3 weeks was started but after 3 administrations, the patient was humanely euthanised 90 days after starting therapy due to progressive disease and the onset of a hind limb paralysis.

## 2 DISCUSSION

Skeletal muscle metastasis (MM) from any primary malignancy is considered very rare (Bar‐Yehuda et al., 2001; Djaldetti et al., 1996; Seely, 1980; Weiss, 1989). This is due to the fact that muscles provide several protective mechanisms against neoplastic invasions, such as accumulation of lactic acid and other metabolites, muscular contractile actions and local pH environment; moreover, the musculature seems able to produce several biochemical anti‐tumoural factors (Bar‐Yehuda et al., 2001; Djaldetti et al., 1996; Seely, 1980; Weiss, 1989). In this report, we described a clinical case of a dog with a suspected diagnosis of NE‐CTCL spreading to skeletal muscle.

In both human and veterinary oncology, muscle metastasis is not considered a common finding, with prevalence reported from 0.03% to 5.6% in autopsy series (García et al., 1984; Hasegawa et al., 2000) and from 1.2% to 1.8% in radiological series in humans (Surov et al., 2014) and 2%–3% in veterinary medicine (Vignoli et al., 2013). However, in a recent report, metastatic lesions to skeletal muscles were reported in the 24.6% of dogs affected by haemangiosarcoma (Carloni et al., 2019), suggesting subclinical metastases to skeletal muscle in dogs could be more common than previously thought.

Skeletal metastasis of lymphoma has been previously reported in a domestic shorthair cat with primary mediastinal lymphoma (Vignoli et al., 2013) and in an Abyssinian cat with an epitheliotropic T‐cell gastrointestinal tract lymphosarcoma (Krecic & Black, 2000). In dogs, muscle involvement in patients with lymphoma has been reported as a primary condition (Fonseca‐Alves et al., 2017; Harkin et al., 2000; Takeuchi et al., 2010; Thuilliez et al., 2008). Interestingly, in two other reports, both muscle and cutaneous lymphomatous lesions were found at the same time in dogs (Baines et al., 2000; Bennett et al., 2005).

As observed here, previously reports describing dogs with muscular invasion had lameness or reluctance to move, however such lesions can also be asymptomatic or result in only vague clinical signs especially in those dogs with primary tumours characterised by a high metastatic rate (Vignoli et al., 2013). This emphasises that whole body computed tomography is preferable for comprehensive staging of oncologic patients.

The small size of the muscle biopsy used for histopathological investigations limited the number of antibodies used for immunohistochemical investigations. Despite this, definitive classification of T‐cell lymphoma was achieved (Valli et al., 2013). Ki‐67 is generally recognised as a useful marker in evaluating the malignancy grade of lymphomas (Fournel‐Fleury et al., 1997) with a cut‐off of 21% or more cells expressing Ki‐67 antigen being considered supportive of high grade lymphomas. On this basis canine cutaneous lymphoma has often been considered as a low‐grade malignancy (Fournel‐Fleury et al., 1997). In human medicine some reports also indicate that proliferation activity in cutaneous lymphomas is lower compared to other T‐cell lymphomas and most probably reflects the indolent nature of these tumours (Kanavaros et al., 2001). On the other hand, one study carried out on heterogeneous group of cutaneous T‐cell lymphomas revealed no differences in Ki‐67 labelling index between different disease entities, regardless of their histological type and grade (Kim et al., 1998), demonstrating that proliferation index cannot be considered a good prognostic marker for these neoplasms. In fact, despite the low Ki‐67 index, the lymphoma investigated in this case report demonstrated aggressive behaviour.

The nodular pattern observed on CT imaging of muscle metastases varies from hypodense with ring enhancement to heterogeneous or homogeneously hyperdense, with or without mineralisation, and various different patterns may be observed within the same patient (Vignoli et al., 2013). In human medicine these different patterns have been classified as type 1 with homogenous contrast enhancement, type 2 with (abscess‐like) hypodense centre and ring enhancement, type 3 inhomogeneous enhancement, type 4 with muscular calcifications and type 5 with intramuscular bleeding defined as an hyperdense area; the most common patterns are the type 1 and 2 (Surov et al., 2014). The dog reported in this case report showed three different patterns, homogenous, heterogeneous and with ring enhancement.

In people, different studies found most MM localised in the trunk musculature, lower extremities and in the gluteal muscles (Haygood et al., 2012), and thigh muscles, extraocular musculature, gluteal and paravertebral muscles (Surov et al., 2014). Interestingly Surov et al. (2014) found that several primary malignancies showed different MM localisations, indeed lung cancer tends to metastasise to the extremities, whereas most MM from breast cancer were located in the extraocular musculature, urothelial carcinomas metastasise more often in the iliopsoas musculature. The explanation could be that the primary tumours have different metastatic routes. Furthermore, it must be presumed that they have different pathophysiological mechanisms of intramuscular metastatic spread (Surov et al., 2014). The most common route of lymphomatous involvement of muscles is metastatic spread from adjacent lymph nodes or other structures (Chun et al., 2010; Suresh et al., 2008). In this case, the involvement of several lymph nodes may have favoured the invasion of more than one muscle compartment, as already described in human cases of lymphoma (Burton et al., 2017; Chun et al., 2010; Gao et al., 2021; Suresh et al., 2008).

In veterinary medicine this differentiation has not been studied yet, however especially in cases of MM from haemangiosarcoma a mixed pattern is often seen (Carloni et al., 2019; Vignoli et al., 2013); therefore, it is not clear if there is a preference of pattern and localisation of the MM for the different tumour types. In the medical literature, three main pathophysiological mechanisms are described. The MM can develop via the arterial route (Surov et al., 2010; Wills, 1953), via venous vessels, especially through the paravertebral venous plexus (Vider et al., 1977), and can originate in intramuscular aberrant lymph nodes, especially MM in the psoas muscle (Lee & Glazer, 1986).

Despite clinical and pathological data suggesting a diagnosis of NE‐CTCL, unfortunately it is not clear if, in this case, the primary lesion was cutaneous rather than a manifestation of a multicentric lymphoma. Nevertheless, as previously reported, lymphoma rarely arises in skeletal muscle, possibly because normal, healthy skeletal muscle does not contain lymphoid tissue (Harkin et al., 2000), suggesting that the nodular pattern in skeletal muscles, observed during TC scan, is consistent with a secondary infiltration rather than primary lesions.

## 3 CONCLUSION

To conclude, this report describes muscular invasion in a case of suspected NE‐CTLC. Although extremely rare, skeletal muscles should be considered as potential location for metastasis of canine lymphomatous neoplasms.

## AUTHOR CONTRIBUTIONS

Conceptualisation: M.V. and P.E.C. Methodology: M.V. Investigation: T.M., M.V., R.T., A.P., F.P., M.S. Data curation: M.V., F.D.S., P.E.C., A.P., A.B. Writing – original draft preparation: T.M., F.D.S., M.V, P.E.C. Writing – review and editing: M.V, R.T., P.E.C., A.P., A.B., A.M. Visualisation: M.V., A.P. Supervision: M.V., P.E.C. All authors have read and agreed to the published version of the manuscript.

## CONFLICT OF INTEREST

The authors declare no conflict of interest.

## FUNDING

This research received no external funding.

## ETHICS STATEMENT

Not applicable because all the information derived from necessary clinical interventions.

## PATIENT CONSENT STATEMENT

For the procedures informed, written consent was obtained from the owner of the animal.

## Data Availability

The data that support the findings of this study are available upon request from the authors.
